# The protective effect of lycopene against oxidative kidney damage associated with combined use of isoniazid and rifampicin in rats

**DOI:** 10.1590/1414-431X2020e10660

**Published:** 2021-05-24

**Authors:** F. Bedir, H. Kocaturk, O. Turangezli, E. Sener, S. Akyuz, F.B. Ozgeris, B. Dabanlioglu, H. Suleyman, D. Altuner, B. Suleyman

**Affiliations:** 1 Department of Urology, Health Sciences University, Erzurum Regional Training and Research Hospital, Erzurum, Turkey; 2 Department of Pathology, Faculty of Medicine, Ataturk University, Erzurum, Turkey; 3 Department of Microbiology, Mengucek Gazi Training and Research Hospital, Erzincan, Turkey; 4 Department of Nutrition and Dietetics, Faculty of Health Sciences, Ataturk University, Erzurum, Turkey; 5 Department of Microbiology, Faculty of Medicine, Erzincan Binali Yildirim University, Erzincan, Turkey; 6 Department of Pharmacology, Faculty of Medicine, Erzincan Binali Yildirim University, Erzincan, Turkey

**Keywords:** Isoniazid, Rifampicin, Kidney damage, Lycopene, Imbalance, Oxidative stress

## Abstract

It is known that the combined use of antibiotics, such as isoniazid and rifampicin, in the treatment of tuberculosis causes oxidative kidney damage. The aim of this study was to biochemically and histopathologically investigate the effect of lycopene on oxidative kidney damage due to the administration of isoniazid and rifampicin in albino Wistar male rats. Lycopene at a dose of 5 mg/kg was orally administered to lycopene+isoniazid+rifampicin (LIR) rats, and normal sunflower oil (0.5 mL) was orally administered to isoniazid+rifampicin (IR) and healthy control (HG) rats as vehicle by gavage. One hour after the administration of lycopene and vehicle, 50 mg/kg isoniazid and rifampicin were given orally to the LIR and IR groups. This procedure was performed once a day for 28 days. Rats were sacrificed by a high dose of anesthesia at the end of this period, and oxidant-antioxidant parameters were measured in the removed kidney tissues. Creatinine and blood urea nitrogen (BUN) levels were measured in blood samples, and kidney tissues were also evaluated histopathologically. The combined administration of isoniazid and rifampicin changed the oxidant-antioxidant balance in favor of oxidants, and it increased blood urea nitrogen and creatinine levels, which are indicators of kidney function. Co-administration of isoniazid and rifampicin also caused oxidative kidney damage. Lycopene biochemically and histopathologically decreased oxidative kidney damage induced by isoniazid and rifampicin administration. These results suggested that lycopene may be beneficial in the treatment of nephrotoxicity due to isoniazid and rifampicin administration.

## Introduction

Tuberculosis caused by Mycobacterium affects not only the lungs but also other vital organs of the body (1). Although tuberculosis is a treatable disease, it is a public health problem representing the second highest cause of mortality among communicable diseases globally (2). Antibiotics, such as isoniazid, rifampicin, pyrazinamide, ethambutol, and streptomycin, are used in the treatment of tuberculosis (3). Although there are various studies in the literature related to the toxicity of anti-tuberculosis drugs on the liver, blood, bone, spleen, and reproductive organs, there are few studies on their nephrotoxic effects (4). However, drug-induced nephrotoxicity is considered to be the cause of 66% of acute kidney failure patients (5). It has been reported that nephrotoxicity due to the combined use of isoniazid and rifampicin is the most important adverse reaction (6). Martin and Sabina reported that the combination of isoniazid and rifampicin causes kidney dysfunction and damage (7). Mahmoud et al. (8) reported that the use of isoniazid and rifampicin causes oxidative damage in kidney tissue. In addition, it has been found that damage to kidney tissues is decreased by antioxidants, which decrease the end products of lipid peroxidation (LPO) such as malondialdehyde (MDA) (7). Together, these studies suggest that the use of antioxidants may be beneficial in the prophylaxis of kidney damage associated with the combined use of isoniazid and rifampicin.

Lycopene is an antioxidant pigment from the carotene family naturally found in vegetables and fruits, and 85% of lycopene is found in tomato and tomato products (9). Lycopene has been shown to reduce oxidative stress (10) and protect the critical biomolecules of cells, including lipids, lipoproteins, and DNA against oxidation (11). Yilmaz et al. (12) showed that lycopene protects kidney tissue from oxidative damage.

However, there are no studies on the protective effect of lycopene against the combined action of isoniazid and rifampicin nephrotoxicity in the literature. The aim of our study was to biochemically and histopathologically investigate the effect of lycopene on oxidative kidney damage caused by the combined use of isoniazid and rifampicin in rats.

## Material and Methods

### Animals

A total of 18 male albino Wistar rats weighing between 277 and 286 grams were randomly selected for the experiment. These animals were obtained from Ataturk University Medical Experimental Application and Research Center (Turkey). The animals were housed and fed at normal room temperature (22°C) for one week before the experiment. Experiments were performed in accordance with the National Guidelines for the Use and Care of Laboratory Animals and were approved by the Animal Ethics Committee of Ataturk University Erzurum, Turkey (Ethics Committee #25.10.2019-75296309-050.01.04-E.1900304439).

### Drugs

Thiopental sodium was supplied by IE Ulagay (Turkey), and isoniazid and rifampicin were supplied by Kocak Farma Pharmaceutical and Chemical Industry (Turkey). Lycopene was supplied by Solgar (USA).

### Experimental groups

Experimental animals were divided into three groups as follows: healthy control (HG), isoniazid+rifampicin (IR), and lycopene+isoniazid+rifampicin (LIR).

### Experimental procedure

Lycopene at a dose of 5 mg/kg was orally administered to LIR rats (n=6), and normal sunflower oil (0.5 mL) was orally administered as vehicle to IR (n=6) and HG (n=6) rats by gavage. One hour after the administration of lycopene and vehicle, 50 mg/kg isoniazid and rifampicin were given orally to the LIR and IR groups. This procedure was performed once a day for 28 days. Rats were then sacrificed by a high dose of anesthesia (50 mg/kg thiopental sodium), and kidney tissues were removed. Thiobarbituric acid reactive substances (TBARS), total glutathione (tGSH), total oxidant status (TOS), and total antioxidant status (TAS) were measured in the removed kidney tissues. Creatinine and blood urea nitrogen (BUN) levels were measured in blood samples. Kidney tissues were also examined histopathologically. Biochemical results obtained from the LIR and HG groups were compared with the results obtained from the IR group.

### Biochemical analyses

TBARS measurement was performed according to the method of Ohkawa et al. (13) based on the principle of measuring the absorbance of the color formed by reactive substances with thiobarbituric acid in a hot acidic environment at 532 nm. In brief, 25 µL of sodium dodecyl sulfate (80 g/L) and 1 mL of acetic acid (200 g/L acetic acid in 1.5 mL of 8 g/L 2-thiobarbituric acid) were added to a 25 µL sample, and the mixture was heated at 95°C for 60 min. After cooling, the mixture was centrifuged for 10 min at 1864 *g* at 4^o^C. The absorbance of the top layer was measured at 532 nm. The TBARS amount in the sample was calculated from the calibration graph generated using 1,1,3,3-tetraethoxypropane as the standard.

tGSH analysis was performed as described by Sedlak and Lindsay (14). When 5,5′-dithiobis [2-nitrobenzoic acid] (DTNB) is reduced to disulfide sulfhydryl groups, a yellow compound is formed, which is then measured at a wavelength of 412 nm. For the measurement, all samples were initially treated with meta-phosphoric acid at a 1:1 ratio and centrifuged at 2000 *g* for 5 min for deproteinization. Then, 150 µL of a measurement mixture (5.85 mL of 100 mM Na-phosphate buffer, 2.8 mL of 1 mM DTNB, 3.75 mL 1 mM NADPH, and 80 µL of 625 U/L glutathione reductase) was added to 50 µL of supernatant taken from the upper phase. Measurements were performed at 412 nm according to the standard graph generated using L-glutathione oxidized (GSSG, Sigma-Aldrcih G4376, USA).

### Measurements of TOS and TAS

Quantitative determination of serum creatinine was performed by a spectrophotometric method using a Cobas 8000 analyzer (Roche Diagnostics, Germany). This kinetic colorimetric test is based on the Jaffe method. Creatinine forms a yellow-orange colored complex with picrate in alkaline solution. This complex was measured at a wavelength of 505 nm. The proportion of colored formation is proportional to the creatinine concentration in the sample. “Rate-blanking” was used in the test to minimize the interference of bilirubin. Serum/plasma results were corrected with -26 mol/L (-0.3 mg/dL) for the nonspecific reaction caused by serum/plasma pseudocreatinine chromogens, including proteins and ketones, as follows: creatinine + picric acid → (alkaline pH) yellow-orange complex.

Quantitative determination of serum urea levels was performed by a spectrophotometric method using a Cobas 8000 analyzer (Roche Diagnostics). BUN levels were calculated using the following formula: BUN = UREA × 0.48. Kinetic testing was performed with urease and glutamate dehydrogenase. Urea is hydrolyzed by urease, and ammonium and carbonate are formed according to the following reaction: urea + 2 H_2_O → (urease) 2 NH_4_^+^ + CO_3_^2-^.

In the second reaction, 2-oxglutarate reacted with ammonium to form L-glutamate when glutamate and dehydrogenase (GLDH) and coenzyme nicotinamide adenine dinucleotide (NADH) are present in the medium. In this reaction, two moles of NADH are oxidized to NAD+ for each mole of hydrolyzed urea as indicated in the following reaction: NH_4_^+^ + 2-oxoglutarate + NADH → (GLDH) L-glutamate + NAD^+^ + H_2_O. The rate of decrease in NADH concentration is directly proportional to the urea concentration in the sample, which was measured at a wavelength of 340 nm.

### Histopathological examination

Removed kidneys were fixed in a 10% neutral buffered formaldehyde solution for 24 h. After routine tissue procedures, 5-µm-thick sections were cut from the prepared paraffin sections and stained with hematoxylin-eosin (H&E) and periodic acid-Schiff (PAS) dye. Histopathological examination was performed under a light microscope (Olympus BX 51, Japan). The cortex and medulla were evaluated in terms of pathological changes in all groups. The images were acquired using a ZEISS Axiocam ICc 5 digital camera (Germany).

### Statistical analyses

Experimental data are reported as means±SE. The significance of the difference between the groups was determined by one-way ANOVA. Fisher's *post hoc* least significant differences (LSD) test was also performed. All statistical analyses were performed using SPSS for Windows (v18.0, IBM, USA), and a P value <0.05 was considered statistically significant.

## Results

### Biochemical findings

**Figure 1:**
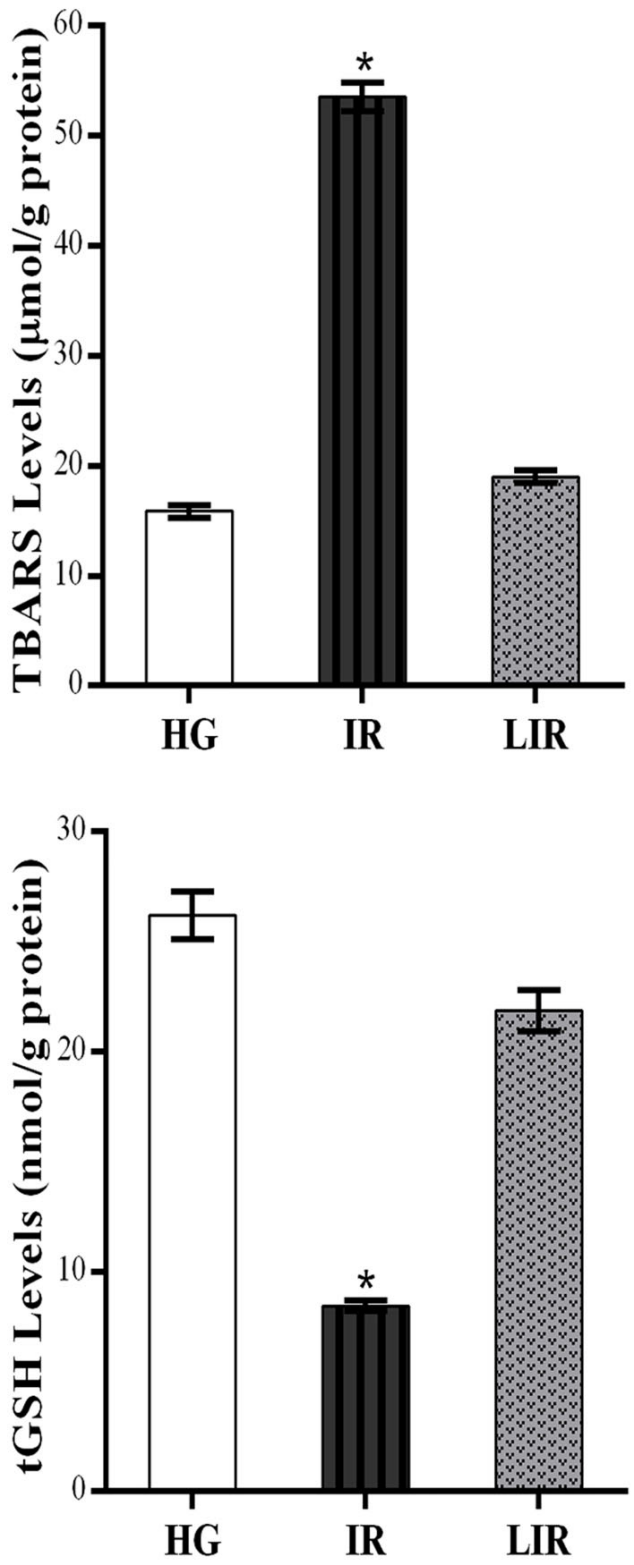
Thiobarbituric acid reactive substances (TBARS) and total glutathione (tGSH) levels of healthy control (HG), isoniazid+rifampicin (IR), and lycopene+isoniazid+rifampicin (LIR) groups in the kidney tissue. Data are reported as means±SE. *P<0.0001 compared to HG and LIR groups (n=6) (ANOVA followed by Fisher's *post hoc* least significant differences).

TBARS levels in the kidney tissues of animals treated with the isoniazid and rifampicin combination increased significantly compared to those of the healthy and lycopene groups (P<0.0001). The difference in TBARS levels between the healthy group and the lycopene group was not statistically significant (P>0.05). In addition, the amount of tGSH in the kidney tissues of animals treated with the isoniazid and rifampicin combination decreased significantly compared to the healthy and lycopene groups (P<0.0001). The difference between the healthy group and lycopene group was not statistically significant (P>0.05) (Figure 1).

**Figure 2:**
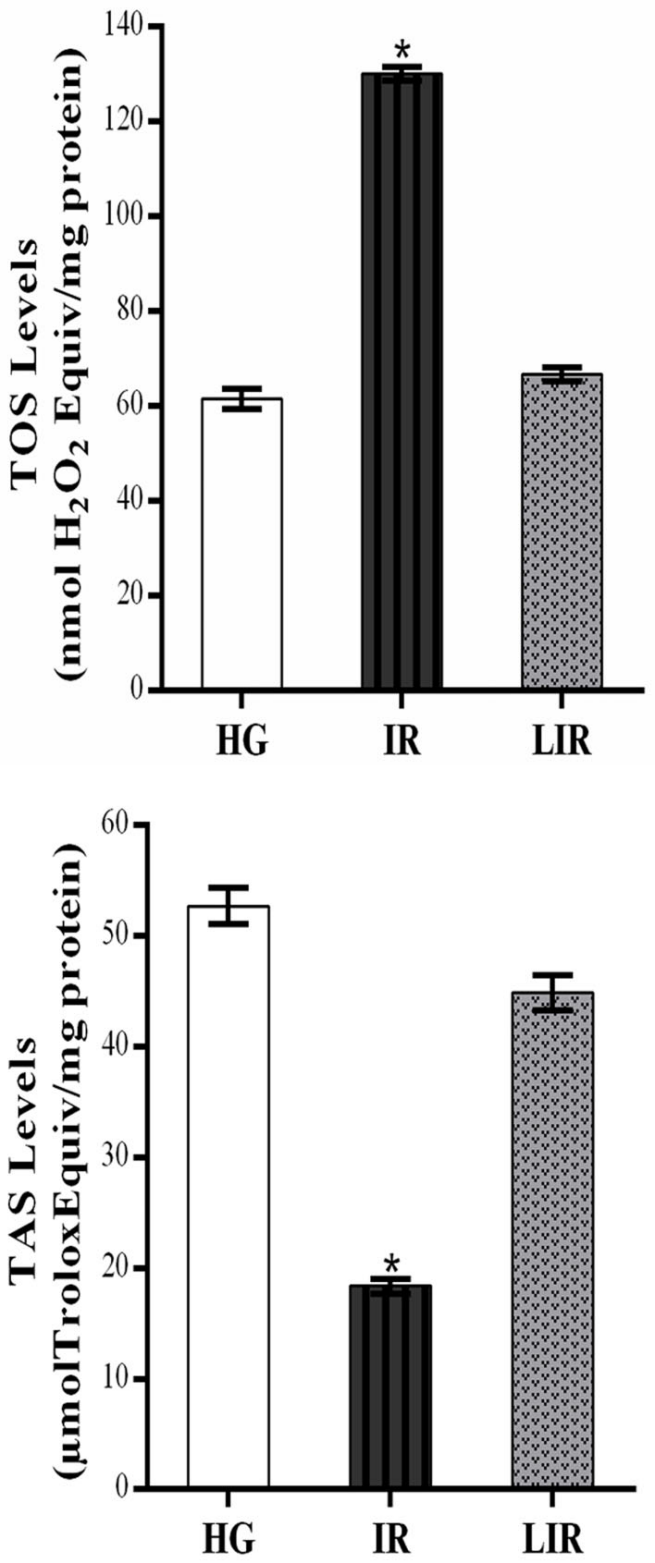
Total oxidant status (TOS) and total antioxidant status (TAS) levels of healthy control (HG), isoniazid+rifampicin (IR), and lycopene+isoniazid+rifampicin (LIR) groups in the kidney tissue. Data are reported as means±SE. *P<0.0001 compared to HG and LIR groups (n=6) (ANOVA followed by Fisher's *post hoc* least significant differences).

TOS levels were significantly increased in the kidney tissues of animals treated with combined isoniazid and rifampicin compared to the healthy and lycopene groups (P<0.0001), while TAS levels decreased significantly. The difference between TOS and TAS levels was not statistically significant in the healthy and lycopene groups (P>0.05) (Figure 2).

**Figure 3:**
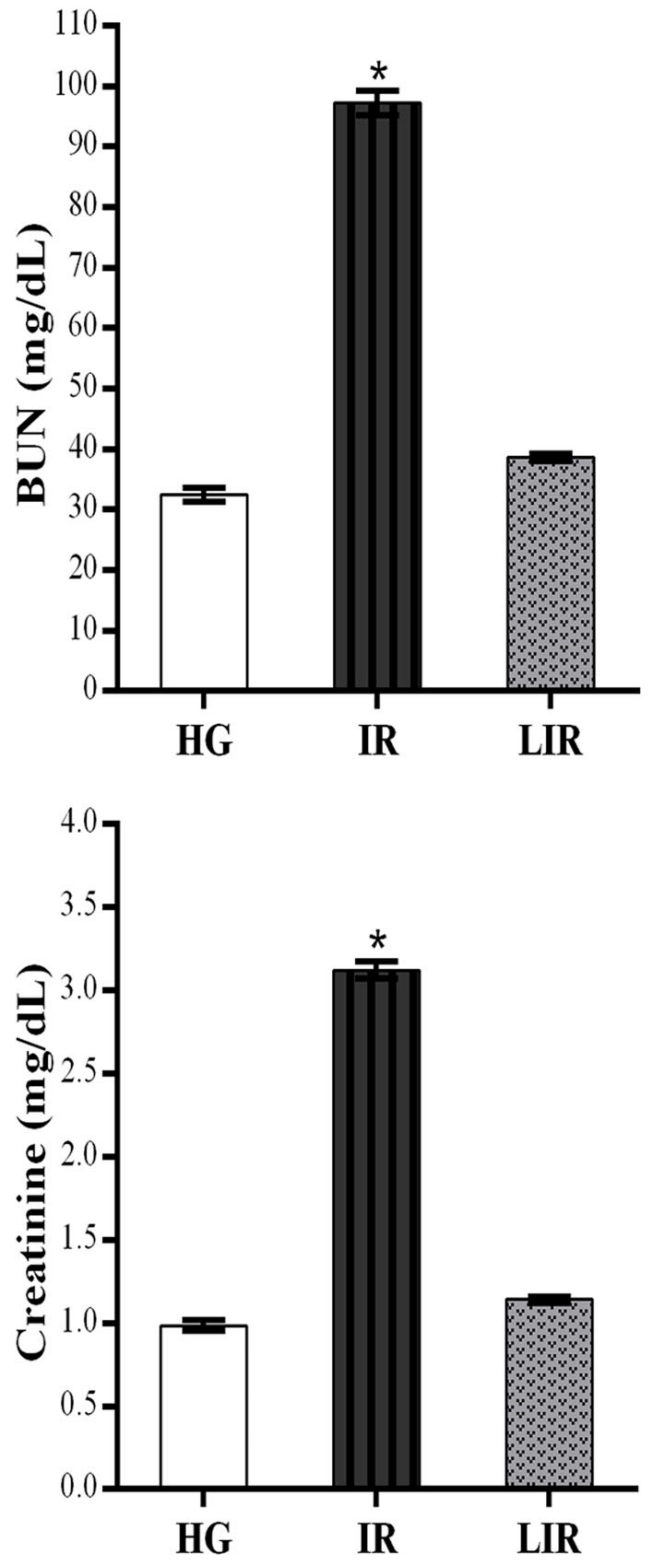
Blood urea nitrogen (BUN) and creatinine levels of healthy control (HG), isoniazid+rifampicin (IR), and lycopene+isoniazid+rifampicin (LIR) groups in the blood samples. Data are reported as means±SE. *P<0.0001 compared to HG and LIR groups (n=6) (ANOVA followed by Fisher's *post hoc* least significant differences).

As shown in Figure 3, the creatinine and BUN levels significantly increased in the blood samples of animals administered the combination of isoniazid and rifampicin compared to the healthy and lycopene groups (P<0.0001). The difference in creatinine and BUN levels between the lycopene and healthy groups was not statistically significant (P>0.05).

### Histopathological findings

**Figure 4:**
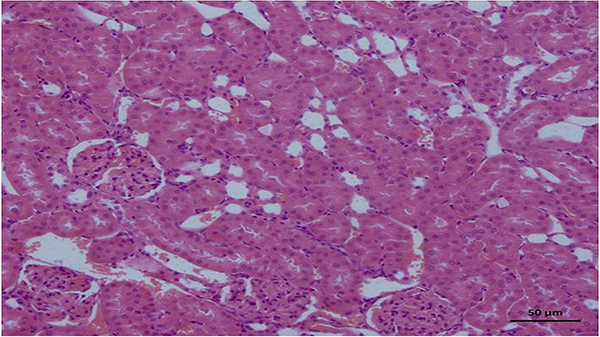
Kidney section (H&E) of the healthy control group showing normal kidney morphology. Scale bar 50 μm.

**Figure 5:**
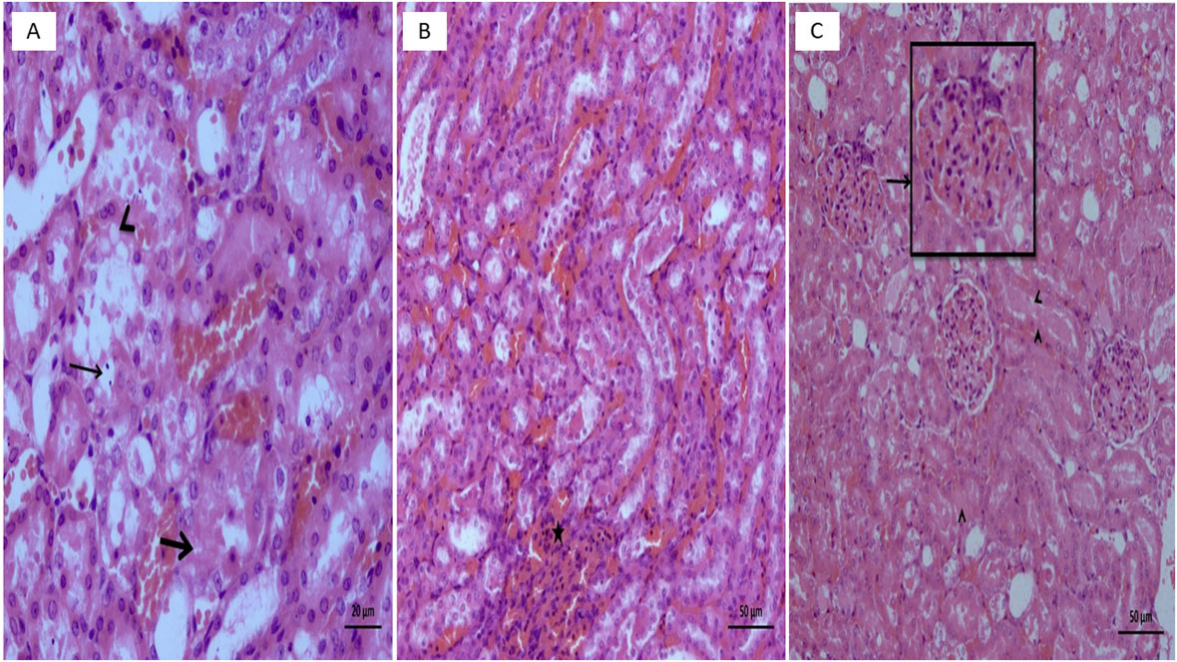
**A**, Necrosis in tubule epithelial cells (thick arrow), vacuolization in tubule epithelial cells (arrowhead), and section showing apoptotic bodies (thin arrow) in the group given isoniazid + rifampicin. **B**, Section showing hemorrhage (asterisk) in the interstitial area in the group given isoniazid + rifampicin. **C**, Section showing intratubular protein cast formation (arrowheads) and congestion (arrow) in the glomeruli in the group given isoniazid + rifampicin (H&E). Scale bar 50 μm.

**Figure 6:**
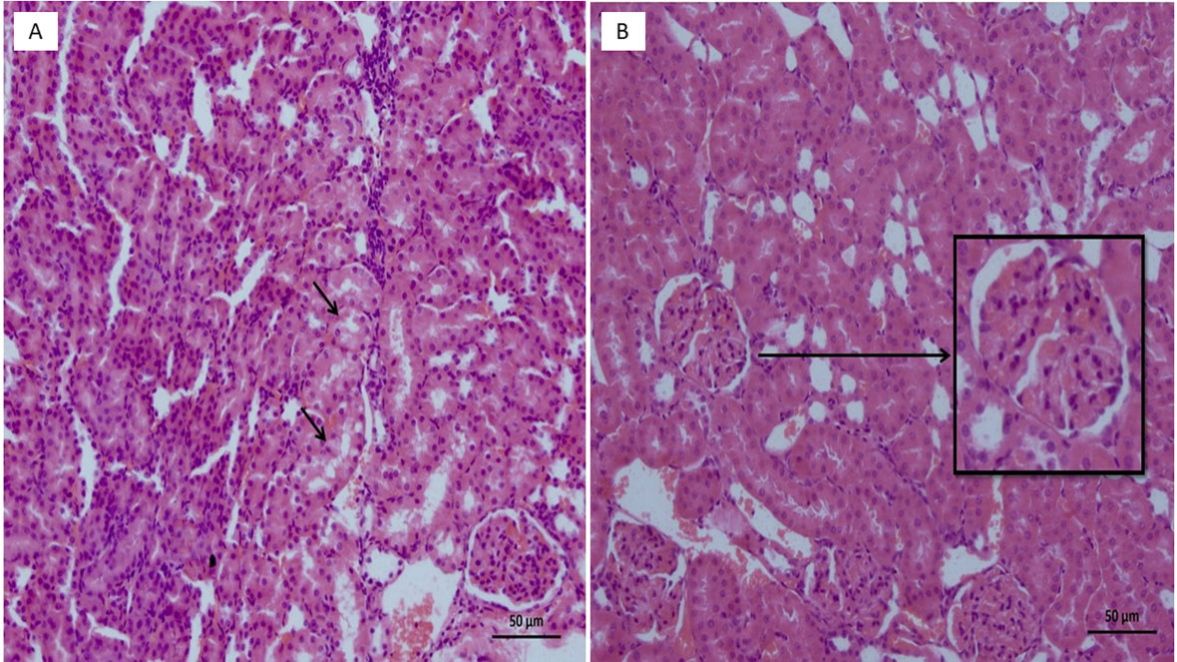
**A**, Section showing mild swelling of tubule epithelial cells (arrows) and **B**, section showing minimal glomerular congestion (arrow) in the isoniazid + rifampicin + lycopene group (H&E). Scale bar 50 μm.

**Figure 7:**
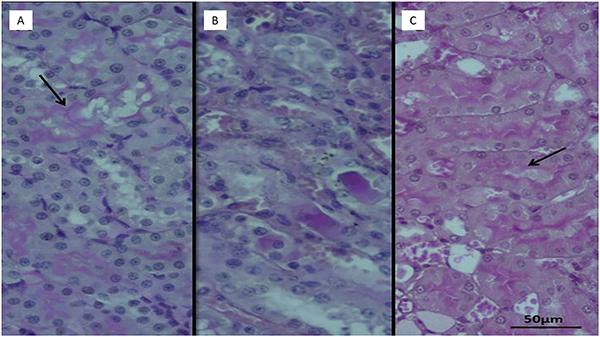
Sections showing normal brush borders (arrows) in proximal tubular epithelial cells in **A,** healthy control group and in **C,** isoniazid + rifampicin + lycopene group (H&E). **B**, Section showing brush border loss due to tubular damage in isoniazid+rifampicin group (PAS staining). Scale bar 50 μm.

No pathological findings were observed in the kidney tissues of the healthy control group, in which normal sunflower oil was utilized as the vehicle (Figure 4). As shown in Figure 5A, however, necrosis and vacuolization in tubular epithelial cells as well as apoptotic bodies and hemorrhage (Figure 5B) in the interstitial area were observed in the kidney tissues of rats administered the combination of isoniazid and rifampicin. In addition, the combination of isoniazid and rifampicin caused intratubular protein cast formation and glomerular congestion (Figure 5C). However, lycopene significantly reduced the pathological damage caused by the combined administration of isoniazid and rifampicin. In the lycopene group, light swelling in tubular epithelial cells (Figure 6A) and minimal glomerular congestion were observed (Figure 6B). In the histopathological examinations using PAS dye, normal brush borders were observed in the proximal tubular epithelial cells in the healthy control (Figure 7A) as well as the LIR group (Figure 7C). The isoniazid and rifampicin combination caused brush border loss due to damage to kidney tubules (Figure 7B).

## Discussion

Nephrotoxicity is an adverse reaction to many drugs, including antituberculosis treatment (15). Rifampicin and isoniazid are antimicrobial agents used solely or in combination in standard antituberculosis therapy (3). According to the literature, the incidence of rifampicin-induced kidney damage ranges from 1.8 to 16% of all acute kidney failure cases and results in 18% mortality (15). In our study, isoniazid and rifampicin applied in combination to induce nephrotoxicity caused an increase in TBARS and TOS levels as well as a decrease in tGSH and TAS levels in animal kidney tissues. These findings indicated that the combination of isoniazid and rifampicin created oxidative stress in animal kidney tissues. The oxidant/antioxidant balance is maintained in healthy tissues with antioxidants being in favor. Various aggressive factors leading to tissue damage change the oxidant/antioxidant balance in favor of oxidants. This phenomenon is known as oxidative stress in the literature (16). Increased TBARS was used to evaluate oxidative stress in the kidney tissues of animals treated with isoniazid and rifampicin in combination. It has been reported that the amount of MDA increases in parallel with the increase in reactive oxygen radicals (ROS) in kidney damage (17). MDA is the end product of ROS-mediated LPO and is the oxidant causing further destruction (18). In another study similar to ours, it was noted that the combination of isoniazid and rifampicin significantly increased the amount of MDA in kidney tissue and decreased the levels of GSH and other antioxidants (7).

GSH is a tripeptide consisting of glutamate, glycine, and cysteine. GSH is an important antioxidant found mostly in the thiol structure at intracellular concentrations (19), and it plays an important role in the detoxification of hydrogen peroxide and free radicals (20). High levels of tissue TBARS and TOS as well as low levels of tGSH and TAS are indications that the balance between all enzymatic and non-enzymatic oxidants/antioxidants in kidney tissue has changed in favor of oxidants. TOS is used to determine the cumulative oxidative effects of various oxidants in biological systems (21), while TAS is used to determine the total antioxidant capacity of different antioxidant molecules (22). The balance between oxidant and antioxidant capacity indicates the sensitivity of organs and tissues to oxidative stress (23).

In the present study, creatinine and BUN levels were increased in the blood serum of the isoniazid and rifampicin groups, in which the oxidant levels were high but the antioxidant levels were low. The kidneys are vital organs for the maintenance of homeostasis, detoxification, and excretion of drugs and toxic metabolites (24). Therefore, kidneys are sensitive to drug-related toxicity. Drug-induced nephrotoxicity occurs as a direct or indirect consequence of exposure to drugs (25). Studies have reported that ROS and oxidative stress play a key role in the pathogenesis of drug-induced kidney damage (26). Martin and Sabina (7) reported that INH + RIF increases creatinine and BUN levels but decreases antioxidant levels. Ramalingam et al. (27) demonstrated that creatinine and BUN levels significantly increase oxidative stress and result in severe kidney failure.

In our study, the levels of TBARS, tGSH, TOS, TAS, creatinine, and BUN in the lycopene group were found to be similar to the levels measured in the healthy control group. To the best of our knowledge, no other study investigating the protective effect of lycopene against nephrotoxicity associated with the use of the isoniazid and rifampicin combination has been reported in the literature. However, it has been reported that 5 mg/kg lycopene reduces aflatoxin-induced nephrotoxicity and cardiotoxicity in animals. Lycopene demonstrates its nephroprotective effect by inhibiting the increase in MDA, creatinine, and BUN as well as by reducing enzymatic and non-enzymatic antioxidants induced by aflatoxin (12). Lycopene has been shown to protect the kidney from furan-induced oxidative damage by improving kidney function, reducing MDA production, and increasing antioxidant levels (28). Serum creatinine and BUN levels are used to evaluate kidney function in experimental nephrotoxicity (29). Das et al. (30) also used BUN and creatinine levels to observe the effects of oxidative stress on kidney function in animals and found that these levels increase with oxidative stress. Kidney injury is defined by a 100% increase in serum creatinine level, while kidney failure is defined by a 3-fold increase in serum creatinine level (31).

All of the biochemical experimental results in this study were consistent with the histopathological findings. Necrosis, vacuolization, apoptotic bodies, and hemorrhage in the interstitial area were observed in the kidney tubular epithelial cells of the isoniazid and rifampicin groups, in which the oxidant levels were high but the antioxidant levels were low. In addition, the combination of isoniazid and rifampicin caused intratubular protein cast formation and congestion in glomeruli. However, lycopene significantly reduced the pathological damage caused by the combination of isoniazid and rifampicin. In recent studies, it has been reported that the drug combination causes kidney tubular epithelial cell damage (32). Sharma et al. (4) showed that the anti-tuberculosis drug combination causes serious histopathological damage in kidney tubules and glomeruli but that necrosis is the predominant finding. Sahu et al. (33) showed that the application of isoniazid alone causes oxidative stress in kidney tissue based on biochemical and histopathological findings. It has been documented that rifampicin alone causes congestion of kidney glomeruli, tubular cast formation, focal degeneration of tubular epithelial cells, peritubular congestion, and partial desquamation of tubular epithelial cells (34).

In conclusion, our biochemical and histopathological findings showed that the combined use of isoniazid and rifampicin caused oxidative stress in animal kidney tissue. The combined use of the drugs changed the oxidant-antioxidant balance in the kidney tissue in favor of oxidants, and lycopene significantly prevented this shift. Our experimental results indicated that lycopene may be effective in the treatment of oxidative kidney injury induced by the use of isoniazid and rifampicin.
